# Return Migration as an Asset Maximization Strategy in Retirement

**DOI:** 10.1093/geroni/igab046.724

**Published:** 2021-12-17

**Authors:** Mara Sheftel

**Affiliations:** Penn State University, Brooklyn, New York, United States

## Abstract

Mexican immigrants make up an increasing proportion of the US population 65 and older. Estimating outcomes for this population is complicated by return migration. Due to data limitations, theoretical frameworks and empirical evidence fail to provide clear indication of the economic selection mechanism of return migration, especially at older ages making it difficult to estimate economic determinants of return. Here two waves of data from the US based Health and Retirement Study and the Mexican Health and Aging Study are combined to create a novel dataset that enables a comparison of assets at older ages for those who stay in the US, those who return before age 50 and those who return at 50 and older. Unadjusted results show no difference in total net wealth at older ages between the three groups, with higher business assets among returnees and higher concentration of wealth in home equity among stayers. With evidence of higher inequality among stayers, lower median wealth in Mexico, and asset advantages operating through citizenship, older age return can be interpreted as a means to acquire a higher standard of living in retirement for non-citizen immigrants. Comparing assets between 2000 and 2012 reveal the vulnerability of stayers during the US housing crisis. These findings are novel because they point to return migration as a retirement strategy and expose a source of vulnerability among those Mexican immigrants who remain in the US into older ages.

